# Influence of Long Milling Time on the Electrical Resistivity of Nanocrystalline Ni_2_MnSn Heusler Alloy Obtained by Mechanosynthesis

**DOI:** 10.3390/nano14131156

**Published:** 2024-07-06

**Authors:** Florin Popa, Traian Florin Marinca, Niculina Argentina Sechel, Dan Ioan Frunzӑ, Ionel Chicinaș

**Affiliations:** Department of Materials Science and Engineering, Technical University of Cluj-Napoca, 103-105 Muncii Avenue, 400641 Cluj-Napoca, Romania; traian.marinca@stm.utcluj.ro (T.F.M.); niculina.sechel@stm.utcluj.ro (N.A.S.); dan.frunza@ipm.utcluj.ro (D.I.F.); ionel.chicinas@stm.utcluj.ro (I.C.)

**Keywords:** Heusler alloys, mechanical alloying, nanocrystalline, lattice strain, electrical resistivity

## Abstract

A Ni_2_MnSn Heusler alloy was obtained as a single B_2_ phase after 12 h of mechanical milling. The influence of prolonged milling on the phase stability was analysed for milling times up to 50 h, related to mean crystallite size, lattice strain, and electrical resistivity. The nature of the powders in the milled range was found to be nanocrystalline, with a mean crystallite size of about 33 ± 2 nm. An evaluation of the internal stresses induced by milling was performed, a linear behaviour was found, and a coefficient of the internal stress increase with milling time was proposed. Particle size distributions of milled samples were analysed, and the morphology of the powders was visualised by scanning electron microscopy. The elemental distribution of milled samples was quantified by energy-dispersive X-ray spectroscopy. Electrical resistivity measurements were performed on compacted samples, and their behaviour with milling time was analysed.

## 1. Introduction

In the past years, a class of materials has gained increasing attention due to multiple properties that they manifest: the Heusler alloys. These alloys extract their properties from a unique combination of three elements combined in a complex structure, the ordered L_21_ structure [1]. The L_21_ structure can be described as a complex face-centred (FCC) structure, where the sites are occupied by the ratio between the valence electrons of the various metals [2]. This structure allows a crystallographic transition from FCC to a martensitic state, a transition close to room temperature [3,4], a magnetic transition almost at almost the same temperature [5], and a shift in electrical resistivity [6]. The combination of the constituent elements and structure gives a unique property for these materials, namely half metallicity, leading to several application opportunities as spintronics materials, magnetic shape memory alloys, and topological insulators [7].

Several compositions exhibit magnetism with a Curie temperature close to room temperature [8,9]; one of them, containing Mn, additionally possesses a large magnetic moment of about 4 μB [10]. This combination occurs in the Ni_2_MnSn Heusler alloy, where the atomic position in the crystal structure is the following: nickel occupies the Wyckoff positions 4a (000) and 4b (1/2,1/2,1/,2), manganese the 4c (1/4,1/4,1/4) site, and tin the 4d (3/4,3/,4,3/4) site [11].

Alongside the magnetic properties, shape memory is another interesting property of the Ni_2_MnSn Heusler alloys. Shape memory alloy properties descriptions were realised by structural commutations, indicating that a small amount of Sn or other metal impurification can provide good properties for usage in the field [12]. Several other practical properties for the Ni-Mn-Sn shape memory alloys were described in [13].

To obtain a Ni_2_MnSn Heusler alloy, the first approach is to melt the composition and perform long-term and high-temperature annealing [11,14], but several other methods are used. Among the used methods, we can cite rapid quenching, which produces alloys with similar crystallographic and magnetic properties as arc melting [15]. Another method used is thin films, with an important application field as devices for spintronics [16].

The interest in the study of the disorder induced by milling in the Ni_2_MnSn alloy is seen in [17], where a cast alloy was crushed by a ball mill, and the effect of particle size and crystallite sizes was analysed from a magnetic point of view. For the milled sample, it was observed that the particles are composed of a small crystallite part surrounded by a large amorphous content [17].

The disorder in the Ni-Mn-Sn Heusler alloys has an important impact on the magnetic properties since the site occupation will differ [18]. For this reason, the stability of the structure was verified by Mossbauer spectroscopy and it was shown that the crystallographic structure of the stoichiometric Heusler alloy is very sensitive to changes in local surroundings, leading to obtaining special materials with desired precipitates able to manifest specific and oriented magnetic properties [19]. As the disorder is introduced, the density of states is also changed [20].

A comparison of the bulk traditional obtaining method with nanocrystalline materials reveals that if we speak about magnetic properties, the magnetisation has lower values compared with the bulk material. When analysing the magnetic ordering, it is found that by switching to a shorter-range order by the introduction of the nanocrystalline aspect, a better tuning of the magnetocaloric effect in these materials can be realised [21].

The electrical resistivity of the Ni_2_MnSn Heusler alloy was found to be dependent on the ordering transition for crystallographic structure and very sensitive to composition, showing that a small variation can shift the values [22].

In the Ni-Mn-Sn Heusler alloys, several studies regarding their acquisition by mechanical alloying can be found [23,24]. For the Ni_2_MnSn alloy, it was shown that by mechanical alloying, the Heusler phase can be obtained after 20 h of milling, but with the disordered B_2_ structure and by using annealing, the L_21_ phase can be promoted [25]. At the same time, if a nonstoichiometric composition is studied, the alloy with a Ni_51_Mn_34_Sn_15_ composition can be obtained after 8–10 h of milling [26].

The interest in obtaining alloys by mechanical alloying (MA) is related to avoiding the melting of the elements. In the arc melting experiments, this is an important issue since Mn is used. In essence, the mechanical alloying technique allows the alloy to be obtained by welding and fracture events repeated throughout the milled period until a very intimate mix of the elements occurs, followed by atom diffusion, and this triggers a reaction process by the energy transferred to the powders at each collision event [27,28].

In this study, we analyse the effect of long milling time on the structure obtained by the solid-state reaction of elemental powders in a planetary ball mill. Aspects regarding the shape and particle sizes are described alongside the elemental distribution maps and, finally, the impact of milling on the electrical resistivity of the Ni_2_MnSn Heusler alloy.

## 2. Materials and Methods

To obtain the Ni_2_MnSn Heusler alloy, elemental powders were used in the stoichiometric ratio. The used powders were purchased from Vale (Ni -Carbonyl type, 3.5–4.5 μm, 99.8 metal basis) and Alpha Aesar (Mn, −325 mesh, 99.3% metal basis, and Sn, −325 mesh, 99.8 metal basis).

The elemental powders mixture was homogenised for 15 min and then milled for times ranging from 12 to 50 h in a Retsch PM200 planetary ball mill. The powder was milled in a 125 mL volume vial, filed with 45 hardened steel balls (10 mm in diameter). The ball-to-powder mass ratio was 15:1. The main disk speed chosen was 300 rpm. During the milling, argon gas was used to protect the powder from oxidation.

The structural characterisations were performed using X-ray diffraction, performed with an INEL Equinox 3000 diffractometer, operating with Co Kα radiation (λ = 1.79026 Å) in the angular range 20–110 degrees.

Using the X-ray diffraction patterns, structural parameters were computed by means of the Rietveld method implemented in the Winplotr software (version April 2023) [29,30]. The Williamson–Hall method was used for the mean crystallite size and lattice strain computation [31,32].

The morphology of the milled powders was analysed by scanning electron microscopy (SEM), performed on a JEOL JSM 5600 LV microscope, equipped with an Energy dispersive X-ray spectrometer (EDX) UltimMAX65 from Oxford Instruments (Aztec 4.2 software).

Particle size distribution was recorded with a Fritch Analysette22 NanoTec in the particle size range 0.1–400 μm in a wet dispersion mode.

The electrical resistivity was measured on pellets of powders obtained by pressing the powders into cylindrical shapes at a pressure of 700 MPa. The samples were measured with four-probe equipment connected to a USB6009 data acquisition board from National Instruments.

## 3. Results and Discussion

**Figure 1:**
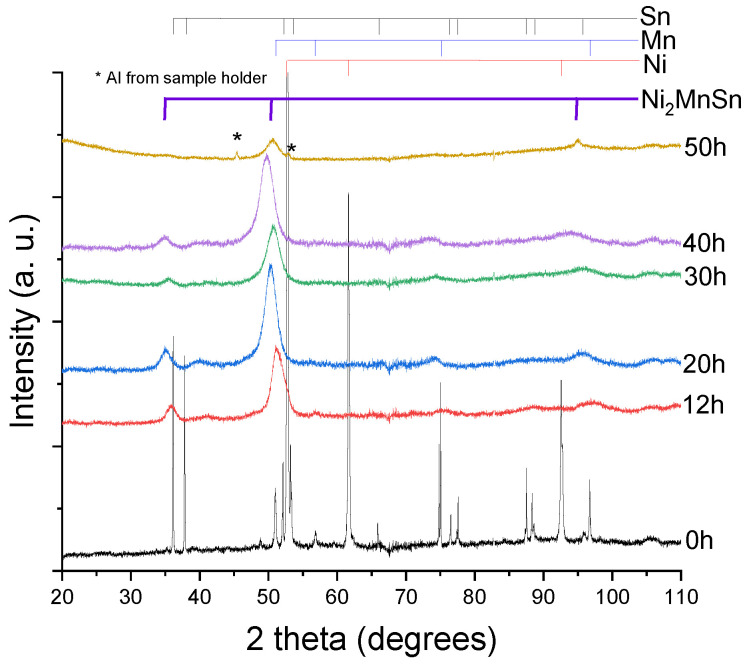
X-ray diffraction patterns for milling times ranging from 12 to 50 h for the Ni_2_MnSn alloy. For comparison reasons, the 0 h (initial elemental powder mixture) milled sample is also given.

Ni_2_MnSn Heusler alloy formation was verified by X-ray diffraction studies for long milling times, as presented in Figure 1.

Compared with the un-milled sample (0 h), the 12 h milled sample shows a different phase composition. If, in the 0 h milled sample, there are visible diffraction peaks for all the elemental components (Ni, Mn and Sn), in the 12 h milled sample, only the Bragg peaks of a Heusler structure are visible, corresponding to the disordered B_2_ structure of the Ni_2_MnSn alloy. The formation of the B_2_ structure is expected to occur in nonequilibrium conditions, according to the phase diagram of the compound, where this phase has the lowest formation energy. Formation time with these types of mills and chosen milling conditions is faster than using other mills [25].

When milling for longer times, up to 50 h of milling, there are no appreciable changes in the phase of the alloy subjected to milling, except for the peaks broadening and the introduction of internal stresses.

**Figure 2:**
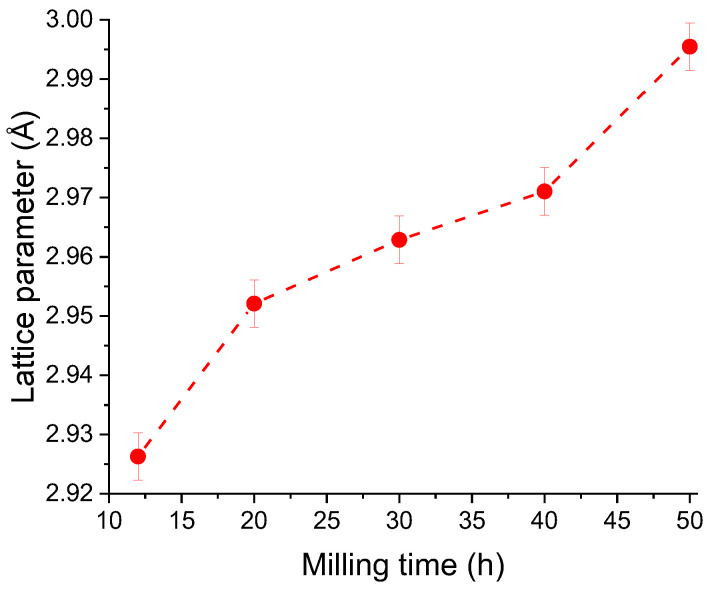
Lattice parameter evolution versus milling time for the Ni_2_MnSn alloy milled for long times.

For these milling times, structural parameters were computed using the Rietveld procedure. The statistical parameters of the Rietveld refinement performed on Ni_2_MnSn milled powders are given in Table 1. Figure 2 presents the evolution of the lattice parameter versus milling time.

The lattice parameter has an increasing trend as the milling is pursued, from a value of 2.926 Å at 12 h of milling to 2.995 Å after 40 h of milling, an increase of about 2.3%. The low milling lattice parameter is comparable with other Heusler alloys with a B_2_ structure [33].

**Figure 3:**
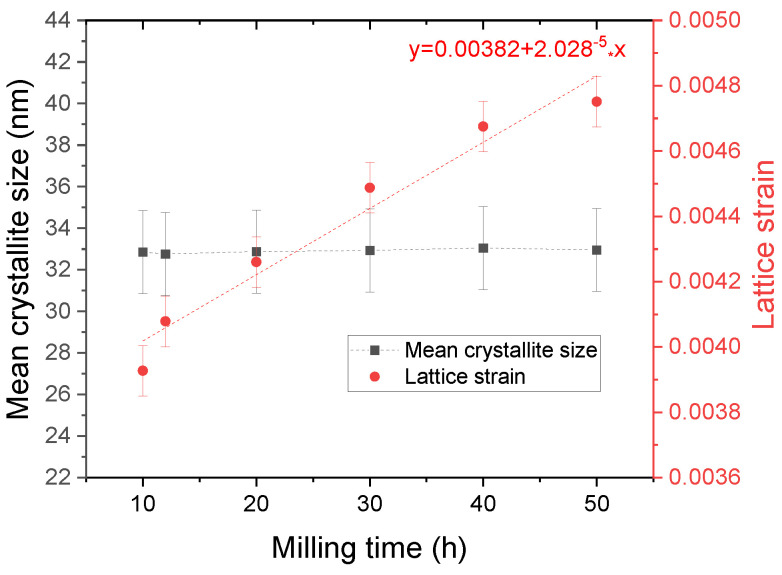
Lattice strain and mean crystallite size dependence with milling time for Ni_2_MnSn Heusler alloy. For the lattice strain, the linear fit used is presented as well.

The increase in the lattice parameter is connected with the increase in the mean crystallite size and lattice strain. The dependence of lattice strains and mean crystallite size is depicted in Figure 3. The mean crystallite size is comparable with other studies of Ni_2_MnSn obtained by mechanical alloying [23]

The lattice strain values increase with the milling time in a linear manner and can be described with a first-order equation. From this equation, it can be concluded that in the acquisition of the Ni_2_MnSn Heusler alloy, there is a minimum amount of lattice strain necessary for the complete reaction of elemental powders by milling. This minimum lattice strain can be derived from fit and is the intercept value of the linear equation, in this case being equal to 0.00382.

Using this linear fit, a variation coefficient of the lattice strain with milling time can be computed by using Equation (1):

(1) $$\alpha =\frac{1}{\varepsilon_{0}}\frac{\Delta \varepsilon }{\Delta t}$$

where α is the lattice strain coefficient with milling, ε_0_ is the lattice strain intercept, and Δε/Δt is the lattice strain variation with milling time.

Using the intercept and the slope, the coefficient of the internal stress accumulation in the powder with milling time increase is equal to 0.00531 (1/h) for the Ni_2_MnSn Heusler compound obtained with these milling conditions.

**Figure 4:**
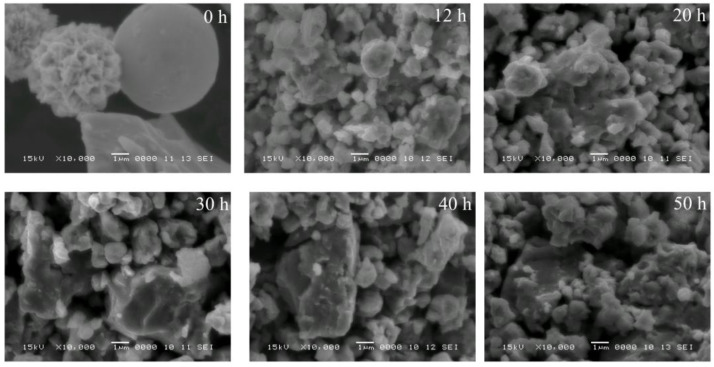
SEM images of the Ni_2_MnSn samples milled from 12 to 50 h. An image of the 0 h milled sample is presented for comparison reasons.

The morphology of the long-milled powders is presented in Figure 4.

**Figure 5:**
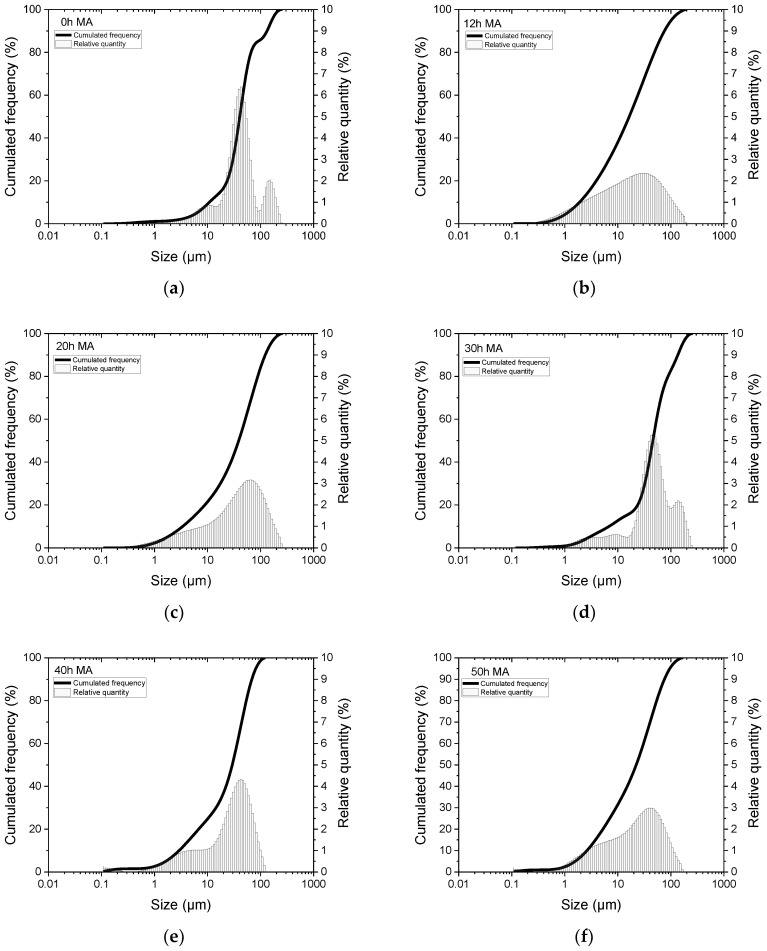
Particle size distribution for the un-milled sample (0 h) and for the samples milled from 12 to 50 h (**a**–**f**).

Looking at the powder morphology, it can be seen that the milling process starts from the big elemental particles of the 0 h milled sample. There is an important refinement up to 12 h of milling, where smaller particles can be seen. In the range of 30 to 50 h, a small increase in the particle size can be observed. The described evolution can be very well evaluated by the particle size analysis. The recorded particle size distributions for several milling times are presented in Figure 5.

Analysing the particle size distribution, it can be seen for the 0 h milled sample the three contributions for the used elemental powders. As the elements react by milling, a large distribution is recorded for the 12 h milled sample. As the Heusler phase is obtained by milling, the particle size distribution shifts, and for 20 h, an increase in the larger particles (about 80 μm) is recorded. For 30 h of milling, the particle size distribution is again shifted toward larger values as the welding process is dominant. For this milling time, three peaks are clearly visible, one for low particle sizes (1–10 μm), a second one at 50–80 μm, and a third one at 120 μm. Milling for even larger milling times (40 h), a two-peak distribution is again recorded, with the main peak at 70 μm. The same type of bimodal distribution is recorded for 50 h of milling, except that the low particle size increases in weight. This behaviour suggests that between 12 and 20 h of milling, the welding processes are the majority processes occurring in the samples, followed at larger milling time by fracture processes. This behaviour can be related to the continuous amount of internal stresses induced in the milled samples by prolonged milling times.

**Figure 6:**
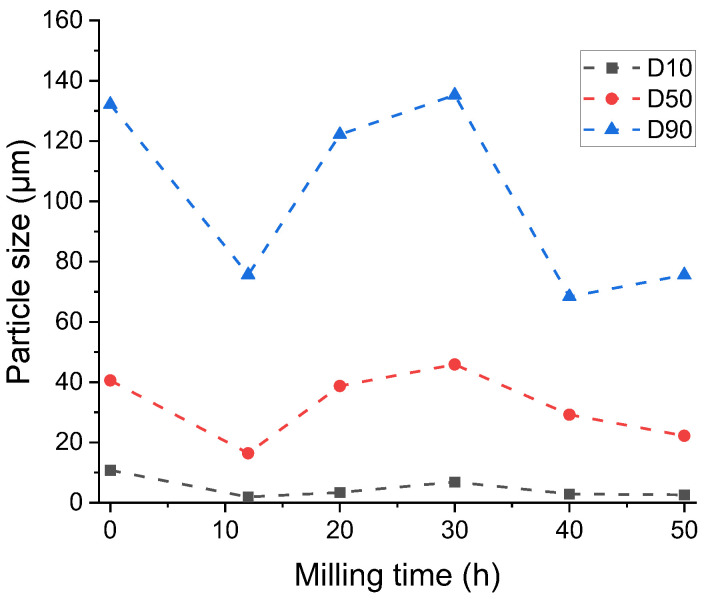
Evolution of the D10, D50, and D90 parameters for the Ni_2_MnSn Heusler alloy milled for long milling times.

To better understand the impact of the particle size distribution, the evolution of the D10, D50, and D90 parameters, meaning the values of the small, medium, and large particle sizes, is presented in Figure 6.

The particle size evolution indicates that for small particle sizes, there is not an important shift as milling is pursued. For medium and large particle sizes, instead, larger variations are present as fractures and then welding and then fracture events again are the dominant behaviours in the powders subjected to milling.

**Figure 7:**
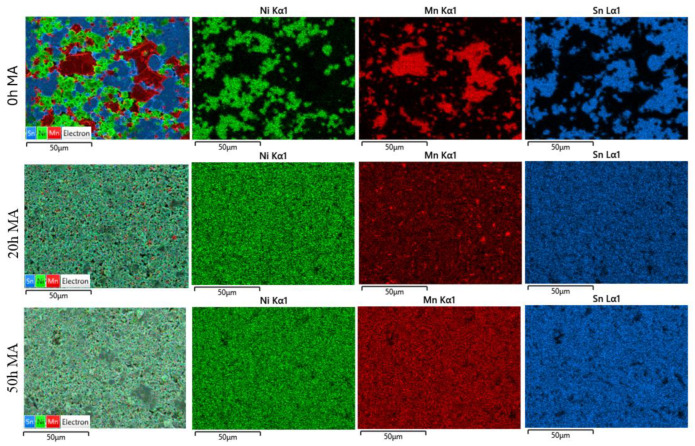
Elemental distribution maps for milled Ni_2_MnSn samples are presented for the Ni, Mn, and Sn elements.

To further understand the formation of the Ni_2_MnSn Heusler alloy and its behaviour under prolonged milling, EDX elemental distribution maps were recorded and are presented in Figure 7 for several milling times.

Looking at the distribution maps of elements, the presence of distinct areas for Ni, Mn, and Sn elements can be clearly seen for the 0 h milled sample. In the 20 h milled sample, some Mn clusters are still visible, although, from X-ray diffraction studies, they were not recorded. Analysing the local composition, it can be concluded that these areas are formed by an alloy with a higher Mn composition than the desired alloy. If the milling is pursued up to 50 h of milling, the size of the Mn clusters is reduced to disappearance, leading to a very nice and homogeneous distribution for all elements, indicating Ni_2_MnSn Heusler alloy formation by milling.

**Figure 8:**
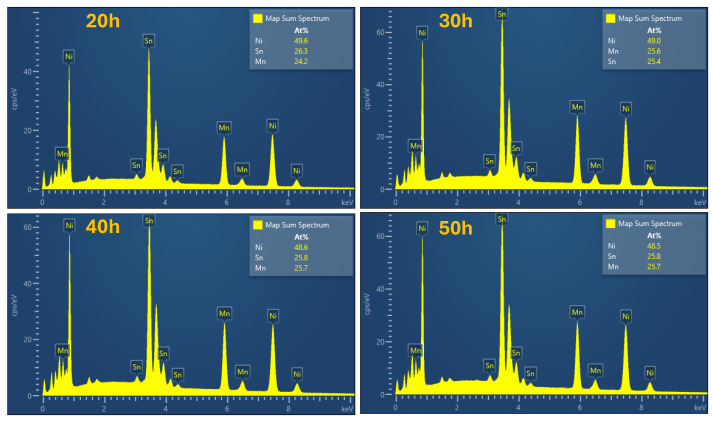
Elemental X-ray energy spectra recorded for Ni_2_MnSn samples milled from 20 to 50 h.

The EDX analysis on the chemical composition indicates a suitable stoichiometry after 50 h of milling, as concluded from the elemental composition analysis based on multiple spectra, as presented in Figure 8 and Table 2 for different milling times.

**Figure 9:**
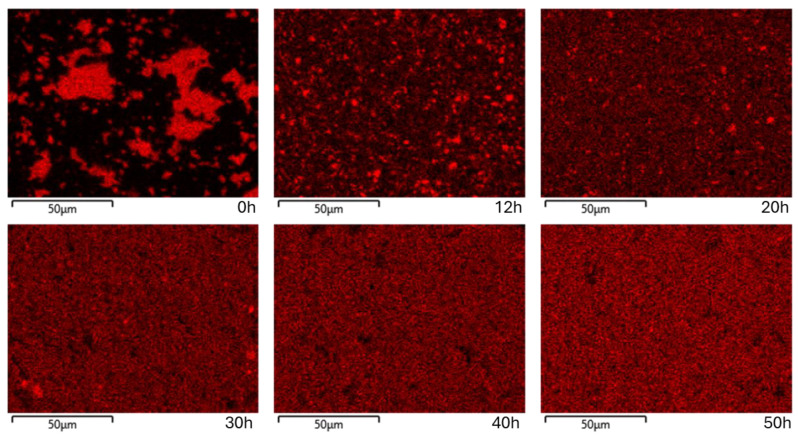
Mn elemental distribution map for Ni_2_MnSn Heusler alloy milled from 12 to 50 h.

A more subtle analysis was performed on the Mn cluster distributions, since in X-ray diffraction, the quantity of unreacted Mn was below the detection limit. Figure 9 depicts the reduction in the Mn clusters as the milling is further continued for prolonged times.

The Mn elemental distribution map shows that this element, although highly reactive with the other two, forms some small clusters, very homogenously distributed after 12 h of milling. For samples milled up to 20 h, the size and number of these clusters are reduced, and this action continues up to 40 h. For samples milled for 40 and 50 h, Mn agglomerations are not found, and it can be presumed that the complete Mn dissolution into the main alloy has occurred. The formation of different precipitates in Ni_2_MnSn alloy was also found in reference [19].

**Figure 10:**
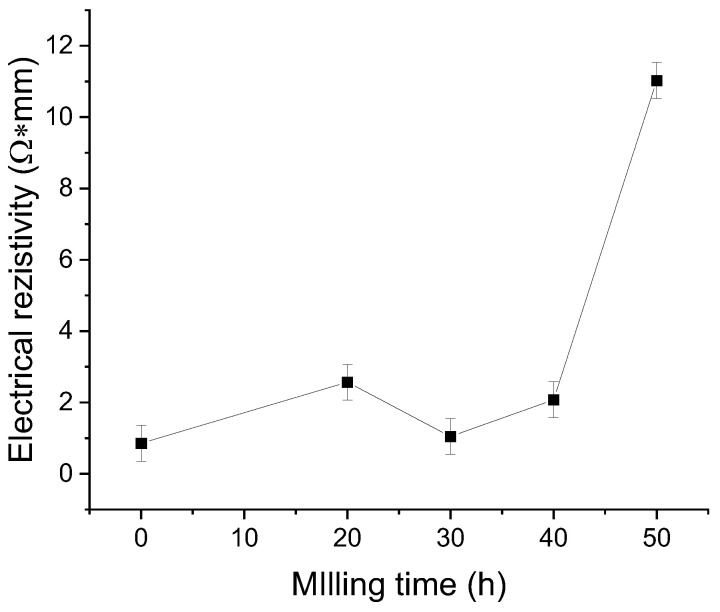
Electrical resistivity evolution of the Ni_2_MnSn Heusler alloy versus milling time.

For the physical properties of the Ni_2_MnSN Heusler alloy, the electrical resistivity was analysed versus milling time. The values of the electrical resistivity versus milling time are presented in Figure 10.

The electrical resistivity of the samples shows a slight increase from the un-milled sample, and it has a relatively small evolution as the milling time increases up to 40 h of milling. From 40 to 50 h of milling, the resistivity has a sharp increase from 2 Ω·mm (40 h) to 11 Ω·mm (50 h). This increase can be related to the disappearance of the last metallic clusters recorded by EDX in the milled samples. Also, the increase at 50 h of milling can be related to possible oxidation of the sample. The obtained values are higher than other reported [34] studies, but generally, the reported values are for the L_21_ structure (ordered) and not for the disordered B_2_ structure, as was obtained in our study.

The acquisition of the alloys in powder shape can offer a technological advance to further use of these materials, since in this form, an increase in the mechanical strength for the Ni_2_MnSn Heusler alloy is achieved [35,36]. By controlling the particle size and compact shape density, the physical properties of the materials can be tailored and adjusted to desired practical values for thermal energy conversion into electricity [1], for example.

## 4. Conclusions

By mechanical alloying, the Ni_2_MnSn Heusler compound was obtained after 12 h of milling. The stability of the obtained compound was verified for up to 50 h of milling. The structure obtained by milling is the disordered B_2_ structure, with a mean crystallite size of 33 ± 2 nm. The lattice strain evolves in a linear way with the milling time, and its evolution can be described by a coefficient equal to a value of 0.00531 (1/h). Elemental distribution maps show that even though the X-ray diffraction patterns indicate the complete formation of the alloy, some Mn clusters are still present in the powders milled for up to 30 h. The particle size distribution exhibits a continuous reduction in particle size as the alloy is milled. Electrical resistivity behaviour versus milling time is influenced by the presence of Mn clusters, leading to an almost constant value of up to 40 h of milling, followed by a sharp increase at 50 h due to alloy homogenisation or by some oxidation that may occur.

## Figures and Tables

**Table 1:** Statistical parameters of the Rietveld refinement performed on Ni_2_MnSn milled powders.

Milling Time (h)	Rp (%)	Rwp (%)	χ^2^
12	3.12	4.04	3.68
20	3.15	4.06	4.78
30	2.92	3.78	2.93
40	3.27	4.13	4.79
50	3.27	4.3	2.12

**Table 2:** Chemical composition of the Ni_2_MnSn sample milled for various times.

Element (at. %)	20 h	30 h	40 h	50 h
Ni	49.6	49.0	48.6	48.5
Mn	26.3	25.6	25.8	25.8
Sn	24.2	25.4	25.7	25.7

## Data Availability

Data are available at request.
